# TUL Herbarium: collections of vascular plants of Tula Oblast, Russia

**DOI:** 10.3897/BDJ.8.e61454

**Published:** 2020-12-24

**Authors:** Tatyana Yu. Svetasheva, Alexey P. Seregin

**Affiliations:** 1 Tula State Lev Tolstoy Pedagogical University, Tula, Russia Tula State Lev Tolstoy Pedagogical University Tula Russia; 2 Lomonosov Moscow State University, Moscow, Russia Lomonosov Moscow State University Moscow Russia; 3 M.V. Lomonosov Moscow State University, Moscow, Russia M.V. Lomonosov Moscow State University Moscow Russia

**Keywords:** Russia, Eastern Europe, Tula Oblast, herbarium, collections, occurrence, specimen, database, digitisation, georeferencing

## Abstract

**Background:**

TUL Herbarium presents collections from Tula Oblast stored at the Tula State Lev Tolstoy Pedagogical University, Russia, which is an educational and scientific institution that supports various types of scientific activities, including research on biodiversity and nature conservation. The university is a holder of some biological collections, such as herbarium of vascular plants, mosses and fungi collected mainly throughout Tula Oblast and from adjacent regions.

**New information:**

The collections of vascular plants (9,000 specimens) were imaged in December 2019 and January 2020. Databasing and georeferencing of the specimens from the TUL Herbarium was performed by the staff members of the Tula State Lev Tolstoy Pedagogical University and Tula Local History Museum. Digital collections of the TUL Herbarium are fully available in the Moscow Digital Herbarium (https://plant.depo.msu.ru/) and GBIF (https://doi.org/10.15468/ca08cm).

## Introduction

TUL is an acronym of the Herbarium of the Tula State Lev Tolstoy Pedagogical University (TSPU), Russia, which is the main custodian of the herbarium collections from Tula Oblast and one of the oldest in Tula institutions with the natural history collections. The acronym was assigned to the Herbarium in 2019 (New York Botanical Garden 2019).

**Geography & Nature**

Tula Oblast is located in the Central Federal District of Russia, covering an area of 25,700 km^2^. It borders Moscow Oblast in the north, Ryazan Oblast in the east, Lipetsk Oblast in the southeast, Oryol Oblast in the southwest and Kaluga Oblast in the west. The climate is moderate continental, with precipitation declining from 575 mm in the northwest to 470 mm in the southeast, average July temperature is about +19...+20°C and average January temperature is −10...−9°C.

Vegetation of Tula Oblast includes zones of broadleaved forests in the central part, forest-steppe in the south and east and a narrow strip of the ecotone subtaiga zone in the west and north. The combination of three vegetation zones contributes to the significant diversity of the flora, which includes both northern and southern elements.

**Historical background**

Tula State Lev Tolstoy Pedagogical University was founded on 19 September 1938 as a federal institution of higher education with the name Tula State Pedagogical Institute. On 18 July 1958, it was named after Lev Tolstoy. On 24 December 1994, it was awarded the status of State Pedagogical University.

The collecting of plant specimens began in the 1960s, when the Faculty of Chemistry and Biology was launched at the Tula State Pedagogical Institute (TSPI). The first collections were made by the students during their summer field practices, as well as from individual assignments on the Botany course. The first herbarium specimens have non-standard appearance and size, since the paper for the specimens was chosen by students at random and it was often cut out manually and then covered with tracing paper attached on the left edge to preserve dry plants. There are ca. 100 those non-standard sheets from the 1960s.

**Figure 1.**
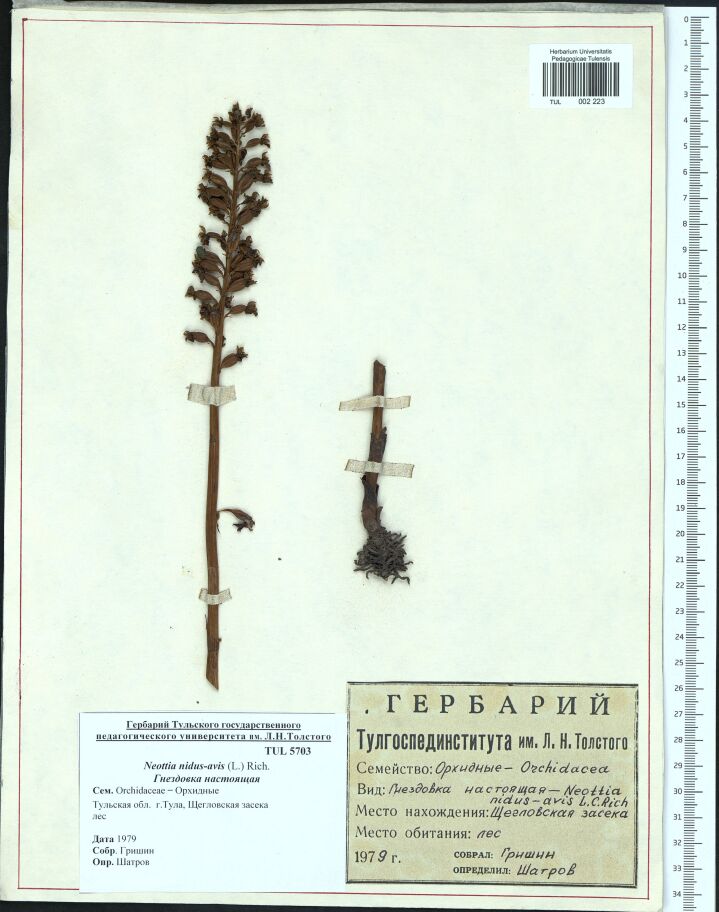
The specimen TUL002223 collected in 1979.

In the 1970s, institute students were the main contributors to the herbarium collections. The specimens acquired a bit more standardised appearance and constant size of sheets 25 x 34 cm, but still differed from the 28 x 42 cm size accepted in the major Russian herbaria. The majority of specimens were supplied with a standard pre-printed label, which included the heading of Tula State Lev Tolstoy Pedagogical Institute in Russian (Fig. 1). With 344 specimens, the total number of collections of 1970s has increased in comparison with the 1960s by about 3.5 times. It is noteworthy that, amongst these old sheets, there are specimens collected by students who later became teachers at the Tula State Pedagogical University and some of them are still working there.

The second half of 1980s became a period of important changes in the scientific life of TSPI. By 1986, the herbarium collection of the Botany Department consisted of several hundred sheets and continued to grow quickly. There was no special hall or even a special cupboard for herbarium storage, so huge piles of specimens were kept just in the labs and staff workrooms.

**Figure 2.**
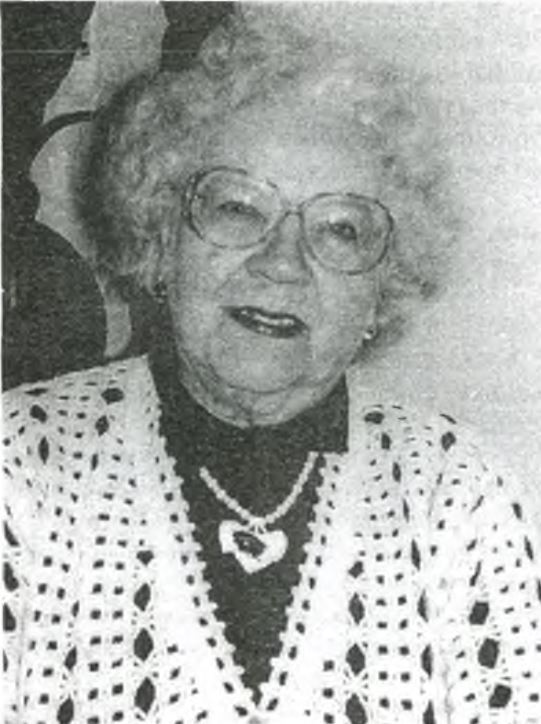
Professor L.F. Tararina, the founder of the Herbarium in Tula State Pedagogical Institute.

**Figure 3.**
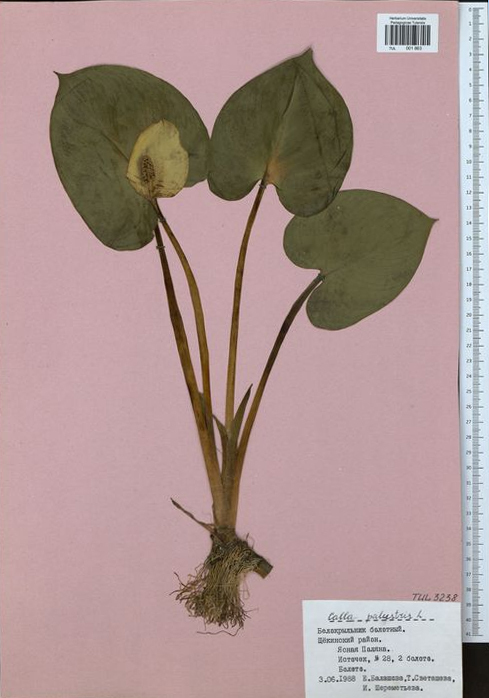
The specimen TUL001863 collected in 1988 (pink paper, 30 x 45 cm)

In 1987, the Botany Department was headed by Professor Lyudmila Fedorovna Tararina (Fig. 2). She initiated the organisation of the scientific herbarium, convinced the administration to provide a room for herbarium storage and equipped it with several custom-made cupboards (Sheremetyeva 2005). In addition, she brought a remarkable feature to the general outlook of our herbarium sheets by ordering a supply of pink paper (30 x 45 cm) for mounting the specimens (Fig. 3).

**Figure 4.**
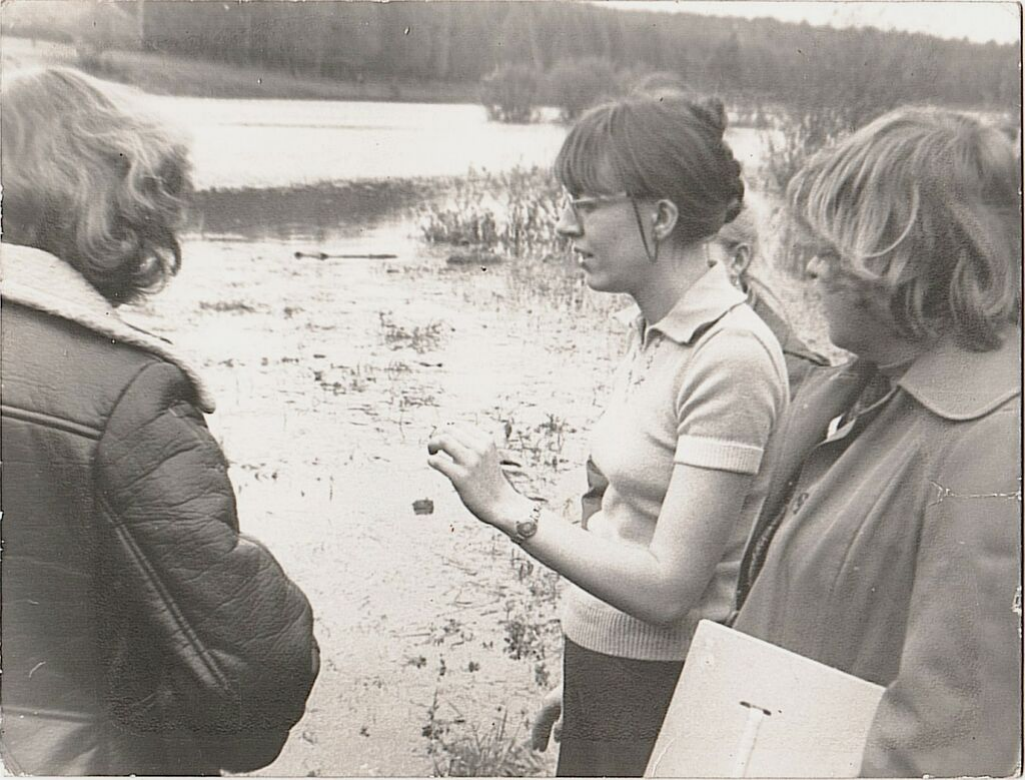
I.S. Sheremetyeva conducting a field practice for students, 1987.

The first curator of the Herbarium in 1987 was Irina Sergeevna Sheremetyeva (Fig. 4), who just at that time started to teach Botany in TSPU and work under her PhD thesis "Flora of Tula Oblast" (Sheremetyeva 1999). The formal foundation of the herbarium gave a powerful impulse to active botanical research. In 1987, 1,368 specimens were collected in a single year, a threefold increase compared to all previous collections. This number includes scientific collections from various districts of Tula Oblast, as well as student's collections made for the scientific herbarium.

**Figure 5.**
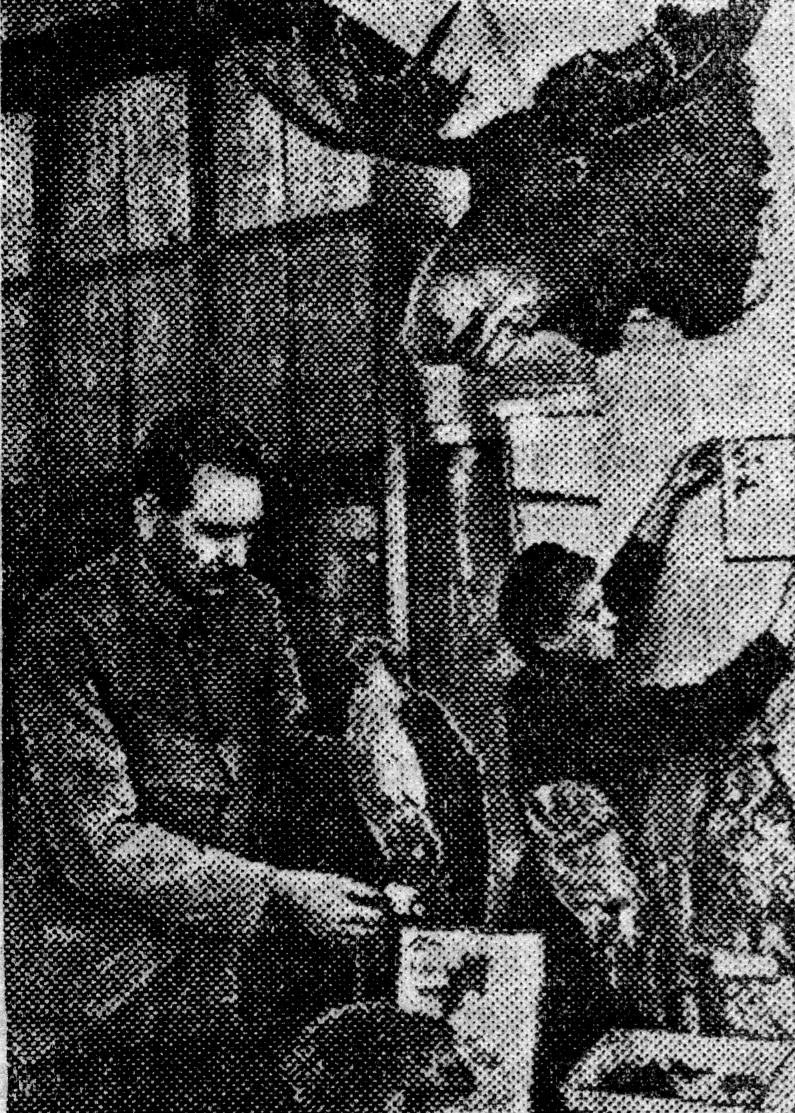
A.I. Alyushin working with zoological collections at the Tula Local History Museum, 1947.

In 1988, a large plant collection of Alexey Ivanovich Alyushin (1897–1987) consisting of 2,104 specimens was gifted by his relatives to the Herbarium. Alyushin was a school teacher and skilled naturalist, who especially loved Botany. In 1941–1945, he also worked at the Tula Local History Museum (Lakomov and Svetasheva 2005) (Fig. 5). He intensively investigated the flora of Tula Oblast and made an outstanding collection of vascular plants. His collections are shared by three Herbaria at present – TUL (2,104 specimens), Tula Local History Museum (ca. 1,000) and MW (297). Alyushin published two popular books about plants of Tula Oblast (Alyushin 1975, Alyushin 1982).

**Figure 6.**
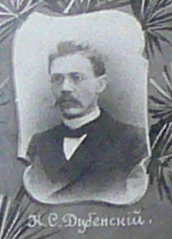
K.S. Dubensky, 1911.

Within Alyushin's collections stored in the TUL Herbarium, there are four specimens of Konstantin Semenovich Dubensky. Dubensky was a teacher of Natural Science at the Tula Real School and Women's Gymnasium in the 1900s–1920s (Fig. 6). He conducted biological excursions in nature and collected plants. His major collections are preserved in the Tula Local History Museum.

Probably, at the same time, the Herbarium received 13 specimens of plants collected by the botanist V.A. Arsenyev in 1931. Unfortunately, the exact information about their origin is unknown.

The period 1987–1999 became the most productive time for the accumulation of plant specimens in the TUL Herbarium (ca. 5,000 specimens). Several scientific research activities, based on intensive collection expeditions across Tula Oblast, were completed at that time, like "Flora of Tula Oblast" (Sheremetyeva 1999), "Adventive flora of Tula Oblast" (Khoroon 1999) and "Register of vegetation cover of Tula Oblast" (unpublished scientific report by L.F. Tararina and I.S. Sheremetyeva). A.V. Shcherbakov from Lomonosov Moscow State University made a large collection of aquatic plants (322 specimens) in line with the preparation of the "Atlas of flora of the Tula Oblast water reservoirs" (Shcherbakov 1999) and habilitation thesis "Hydrophilic flora of vascular plants as a model object for inventory and analysis of flora: the example of the Tula and neighboring regions" (Shcherbakov 2011). Large collections on the flora of Yasnaya Polyana Museum-Estate were made by E.G. Balashova, T.Yu. Svetasheva and I.L. Dorokhina for their master's theses (782 specimens).

In the 2000s, the Herbarium was supplemented with ca. 660 specimens mainly due to a series of works devoted to the study of protected areas of Tula Oblast (1999–2005, under L.F. Tararina) and the preparation of the "Red Data Book: Specially Protected Natural Areas of Tula Oblast" (Tararina et al. 2007) and "Red Data Book of Tula Oblast: Plants and Fungi" (Shcherbakov 2010). Therefore, a significant part of those collections includes specimens of rare and threatened plants. The standard regional checklist, entitled "Synopsis of the flora of vascular plants of Tula Oblast", was published upon thorough study of available collections and vast literature (Sheremetyeva et al. 2008).

After 2010, the Herbarium continued to be filled mainly by the collections of students, but their collections were transferred to the educational collection, which is used during lab classes and is not part of the Herbarium. However, in 2018–2019, the TUL Herbarium was replenished with recently-collected specimens due to the preparation of the second edition of the regional "Red Data Book" and monitoring of the protected natural areas (Sheremetyeva and Svetasheva 2019, Svetasheva and Sheremetyeva 2019, Svetasheva et al. 2019). It should be noted that, since the 2000s, the Tula scientists began to form collections of the other groups, for example, fungi, lichens and mosses. Gradually, by 2019, the TUL Herbarium represents a significant base of the Tula Oblast biodiversity, including ca. 10,000 specimens of vascular plants, ca. 1,000 specimens of bryophytes and ca. 2,000 specimens of fungi and lichens.

Today, the Herbarium occupies one room (ca. 25 m^2^) in the building of the Faculty of Natural Sciences of Tula State Lev Tolstoy Pedagogical University located on 125, Lenina Prospect, in Tula. There is no special regular staff in the TUL Herbarium.

At the end of 2018, Lomonosov Moscow State University, which also houses some plant collections from Tula Oblast, suggested to combine efforts to digitise the entire collection of the Tula flora and place the obtained data on the Internet using a platform of the Moscow Digital Herbarium. The created project "Diversity assessment and visualisation of the Tula Oblast flora using modern data technologies" has been supported by RFBR and Tula Oblast Goverment and this has brought the Herbarium to a higher level. In 2019, the acronym TUL was assigned to the Herbarium of Tula State Lev Tolstoy University after its registration in Index Herbariorum. By 2020, after the first year of project, the larger part of the collections of vascular plants has been digitised, georeferenced and finally published within the Moscow Digital Herbarium and on GBIF.

## Project description

### Title

Diversity assessment and visualisation of the Tula Oblast flora using modern data technologies

### Personnel

Tatyana Svetasheva (Tula State Lev Tolstoy Pegagogical University) ― general management and supervision of the project, preparation of collections for digitisation, databasing of specimens, georeferencing;

Alexey Seregin (Lomonosov Moscow State University) ― project design, general consulting, management and supervision of imaging and other digitisation activities, online publication of the collections, identification of specimens;

Irina Sheremetyeva (Tula State Lev Tolstoy Pegagogical University) ― identification of specimens and verification of earlier definitions, collection of fresh specimens;

Lyudmila Khoroon (Tula State Lev Tolstoy Pegagogical University) ― identification and verification of specimens, preparation of collections for digitisation;

Alexander Lakomov (Tula Local History Museum) ― georeferencing;

Dina Zatsarinnaya (Tula Local History Museum) ― georeferencing, preparation for digitisation of the collections of Tula Local History Museum (in process);

Elena Volkova (Tula State University) ― preparation for digitisation of the collections of Tula State University and State Museum Reserve "Kulikovo field" (in process);

Oleg Platko (Lomonosov Moscow State University) ― online publication of the collections, data audit, database administration.

### Study area description

Tula Oblast

### Design description

**Project Background and Aims.** Biodiversity conservation is an urgent task for mankind. This environmental challenge is particularly acute in the Tula Oblast characterised by a highly-developed industry, a high proportion of agricultural land, as well as by a low proportion of protected natural areas. The project aimed at the inventory of vascular plant diversity at a new level. For the first time in Tula Oblast, a text database and GIS-module with geolocations have been complemented by the Library of high quality images of herbarium collections. The goal of the project is to assess the actual diversity and spatial structure of the Tula flora by combining various data sources on a single platform, visualising all the available information by digitising data and creating a multifunctional electronic resource with an open access. The main scientific idea is to make the Tula flora visible to the global community.

**Project steps.**

1. To combine the data on five herbarium collections made in Tula Oblast (stored in Tula State Lev Tolstoy Pedagogical University (TUL), Tula State University, Tula Local History Museum, State Museum Reserve "Kulikovo field" and Moscow State University (MW));
2. to image herbarium specimens;
3. to supplement the materials with photographs of plants in the wild;
4. to outwork a database of label transcriptions;
5. to georeference every specimen;
6. to create a regional information online resource "Digital Herbarium of Tula Oblast" (ca. 20,000 records).

The project is scheduled to run for three years and includes laboratory work with collections and field studies of the Tula Oblast flora. The first year of the project (from May of 2019 to May of 2020) was devoted to the accumulation and structuring of electronic data on the regional diversity of vascular plants. We performed processing (inventory, taxonomic revision and databasing) and digitisation of plant collections from Tula Oblast, preserved in Tula State Lev Tolstoy Pedagogical University (TUL) and Lomonosov Moscow State University (MW). As a result, 13,931 images made at 300 dpi were published via the Moscow Digital Herbarium (https://plant.depo.msu.ru/), including 9,000 specimens from the TUL Herbarium. We also made transcriptions of 12,991 labels and georeferencing of 12,909 specimens. Finally, the dataset of the TUL Herbarium has been published in GBIF and the dataset of MW has been published separately (Seregin 2020).

The obtained data on the Tula Oblast flora have already been used during the preparation of the second edition of the regional "Red Data Book" (Shcherbakov 2020, in press). These data are a solid basis for compiling general floristic reports, assessing the conservation status of species at various levels and developing the strategies for legal protection of rare species and plant communities.

### Funding

See Acknowledgements section.

## Sampling methods

### Sampling description

**Preparation for digitisation**

The preparation of the Herbarium collection for imaging included several steps.

1. A set of traditional methods and techniques for processing of herbarium specimens (Skvortsov 1977, Geltman 1995), checking the current state of previously-collected specimens, mounting; collecting and complete processing of new accessions.
2. A critical revision of identifications with the involvement of highly-qualified botanists specialising in certain taxonomic groups of plants: S.R. Mayorov — genera *Thymus*, *Oenothera*, *Physalis*, *Sisymbrium*, *Coreopsis*; A.V. Shcherbakov — family Potamogetonaceae; A.P. Seregin — genera *Carex*, *Galium*, *Silene*, *Dactylorhiza*.
3. Full label capturing into the MS Access database. The database consists of a system of interconnected tables and a set of frequently-used queries. The main table contains specimen information (phylum, class, family in Latin and in Russian; genus in Latin; species in Latin; authors of species; infraspecific taxa; authors of the infraspecific taxa; synonym from the original label; species name in Russian; geographical region; administrative region; administrative district; city; toponym (i.e. location, the nearest settlement or its vicinity, natural boundary etc.); habitat; collection date; collector's name; identifier's name; re-identification date; re-identifier's name; notes). The main Table also includes some technical fields for the data processing — sign (any character entered when creating special queries); number of the record (unique number that automatically increases by 1 in each new record); the herbarium number (unique number assigned to a specimen); date of recording; recording person.
4. Quality control. Сlarification and correction of the entered information with the help of specific queries selecting data on a certain collector, date, location etc. Some important information has been found in literature and some unpublished sources of botanists who worked in Tula Oblast. This allowed us to restore some information gaps in the available data and include them in the database.
5. Labelling. Each specimen has two labels, i.e. printed label retrieved from the database and the original one. New labels with the unique specimen number and TUL acronym have been obtained from the database by programming external links between MS Access and MS Word. In addition to the new ones, the original labels made by collectors have been placed on the Herbarium sheets to clarify the historical component of the Herbarium.
6. Barcoding. A barcode contains the name of the TUL Herbarium in Latin (Herbarium Universitatis Pedagogical Tulensis), TUL acronym and a six-digit number (like TUL006192). The barcodes were pre-ordered and printed typographically.

**Creation a library of images (scanning and processing)**

Imaging was carried out by the commercial partner (https://elar.ru/) on planetary scanners of A2 format with a resolution of 300 dpi in two steps in December 2019 and January 2020. The contribution from the commercial partner included:

1. transportation of 9,000 specimens from Tula to Moscow for imaging;
2. imaging of the front sides in TIFF format (300 dpi) with a scale bar;
3. converting TIFF files to JPEG copies;
4. renaming of the files against barcodes;
5. structuring of graphic images in accordance with their physical storage within the herbarium (directory names equal species names on folders);
6. manual quality control of the final images;
7. recording of images on storage media (HDD).

**Electronic data capture**

Each image was supplied with minimum metadata, for example, ID against barcode, species name from the folder and area code (Tula Oblast). The records from the MS Access database with full label transcriptions were cross-linked using barcode IDs and integrated into the dataset.

**Georeferencing specimen data**

Manual georeferencing is carried out using standard e-cartographic libraries (Yandex.Maps, Google Maps etc.). We used the old scanned maps (of the early 20th century) for georeferencing of some historical collections, since the names of some geographic locations have been changed or disappeared over time.

Manual georeferencing was supplemented by automatic georeferencing by the ISTRA system (Intellectual System of Toponymic Reading and Attribution), with several lines of the code being written in JAVA (Seregin and Stepanova 2020). This code is integrated into the Moscow Digital Herbarium and unavailable as a stand-alone product. The first algorithm of the ISTRA system combines the specimens into the groups according to the matching of the captured label text. In this case, there are two options for combining — complete matching mode and letters-only mode. The results do not differ in accuracy from the manual georeferencing. The second algorithm of the ISTRA system forms the specimen groups according to the matching of three fields—collection date, collector’s surname and curatorial area. Within the walking-day route, the standard georeferencing accuracy in most cases does not exceed 5 km. Further data refinement will help us to replace automatic georeferencing with a more accurate manual one. In both cases, the operator inserts the coordinates manually and the system sets the coordinates automatically for all specimens of the group. The first algorithm takes precedence over the second one. We save the log file and note the georeferencing method in the form of the standard disclaimers.

**Publication of digitised collections**

Digitised collections of the TUL Herbarium were published in the Moscow Digital Herbarium. This means that images and metadata are available on three platforms (Seregin 2018).

1. Moscow Digital Herbarium (https://plant.depo.msu.ru/) is actually an operational version with a number of search tools like label search, geosearch, search on the taxonomic tree, search by Latin and vernacular names etc. Data administrators of Lomonosov Moscow State University are managing the content and editing the data. IT staff are incorporating new large datasets like labels, georeferences, taxonomic treatments etc. The content of the portal is hidden from search robots, but users can download XLSX-files with general metadata. One can see basic statistics of the Moscow Digital Herbarium right on the homepage.
2. Open version (https://plant.depo.msu.ru/open/). – This portal is optimised for computers, tablets and mobile platforms. The content is updated once a day. The open version allows search robots to access the text and JPG images, as well as quick simple searches through all fields of the database. The content is accessible 24/7 even if we are updating the system or content. One can see search results in the form of small icons and search criteria could be saved as a unique URL.
3. Global Biodiversity Information Facility (https://www.gbif.org/). – Our data are fully available in GBIF without any limitations (Svetasheva and Seregin 2020). Since 2 November 2017, Moscow Digital Herbarium is updating the GBIF-mirror once a week, including datasets of the consortium members (Seregin 2020a). Loading the data into GBIF helps us check data consistency and find mistakes, like coordinate swapping, inaccurate georeferencing, errors in dates etc. The GBIF portal is the only place where one can download our geodata. The real advantage of GBIF is that it delivers our information to a wider community.

## Geographic coverage

### Description

The TUL Herbarium was initially launched as a regional one and focused on the flora of Tula Oblast, including native and alien plants. Only a small part of the collections represents plant specimens from other regions of Russia and is not yet digitised. The main goal of plant collecting for years was a thorough investigation of various plant communities in all administrative districts of Tula Oblast according to the grid-mapping scheme (Shcherbakov 1999).

However, there are a number of reasons that did not allow complete sampling throughout the area:

- peculiarities of human transformation of vegetation cover of Tula Oblast, including wide distribution of agricultural land;
- more detailed study of protected nature areas;
- restricted access to certain areas (like museum-reserves);
- intensive studies of some areas during summer field practices of students.

**Figure 7.**
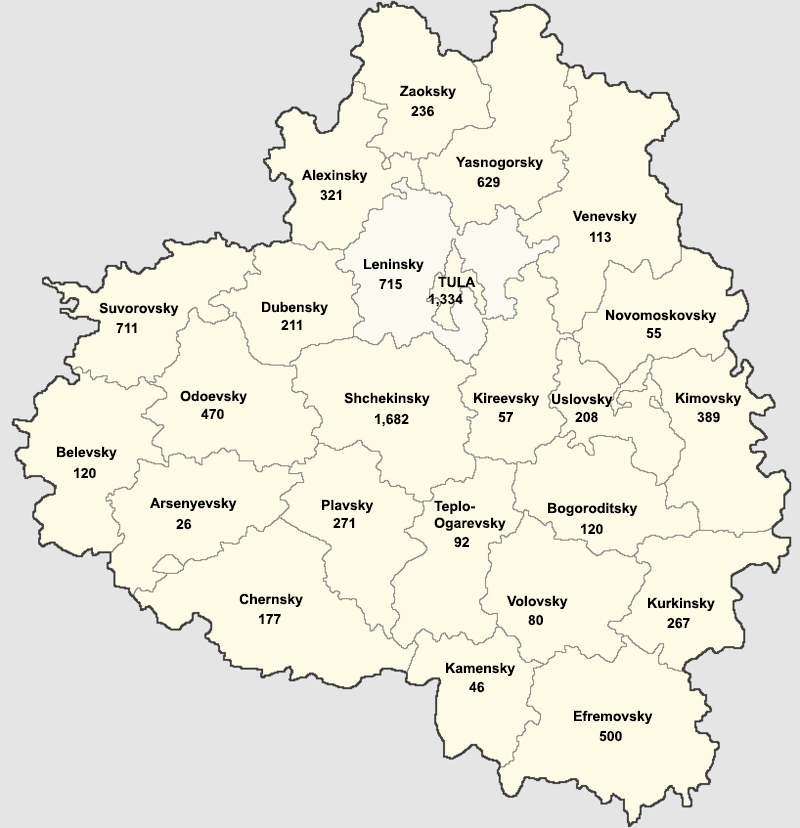
Map of the administrative districts of Tula Oblast. Number of digitised specimens is given for every district in Table 1.

As a result, some districts of Tula Oblast are well-studied and represented by numerous specimens, whereas other districts are poorly sampled. In Table 1, one can see the number of digitised specimens available in GBIF and number of all databased specimens collected in the administrative districts of Tula Oblast (Fig. 7) ranked in descending order.

The most sampled area is Shchekinsky District (1,682 digitised specimens), where Leo Tolstoy Museum-Estate "Yasnaya Polyana" is situated. This institution is involved with nature conservation and biodiversity research and periodically supports studies of various groups of organisms. For example, some student master's theses (for instance, "Birch forests of Yasnaya Polyna" by T. Svetasheva, 1987–1991; "Spruce plantations of Yasnaya Polyana" by E.G. Balashova, 1987–1991) were performed due to the on-purpose contracts between the Museum-Estate and TSPI. From the other side, the Museum-Estate is situated not far from the University and is a convenenient place for field practices and botanical excursions.

The City of Tula (1,334) and adjacent Leninsky District (715) are holding the second and the third positions in the ranking. These administrative units are the places of regular excursions and individual assignments, as well as scientific research of the alien flora.

They are superseded by Suvorovsky, Yasnogorsky, Efremovsky, Odoevsky, Kimovsky, and Aleksinsky districts, where local biodiversity hotspots (including localities of rare and threatened plants), as well as some protected areas, are situated. Other districts are either situated on the fringes of the Tula Oblast or have vegetation greatly affected by human activity.

### Coordinates

52.9 and 54.9 Latitude; 35.9 and 39 Longitude.

## Taxonomic coverage

### Description

Most of the TUL Herbarium collections are represented by vascular plants from Tula Oblast, of which 95% are currently digitised. The focus of the Herbarium is on native and alien flora, whereas cultivated plants of Tula Oblast are almost missing. The brief taxonomic outline of the collections is based on GBIF dataset with 9,000 specimens.

For many years, we followed the standard "Flora of the middle zone of the European part of Russia" by P.F. Mayevsky (Maevsky 2006, Maevsky 2014) for taxonomy and nomenclature. In recent years, with the emergence of powerful online resources, we used for databasing the taxonomic backbone of the "Plantarium: open on-line atlas and key to plants and lichens of Russia and neighbouring countries" (Oreshkin 2020), based on several modern taxonomic sources. In the Moscow Digital Herbarium, all names accepted in the collection were automatically cross-referenced with the Catalogue of Life (https://www.catalogueoflife.org/).

**Figure 8.**
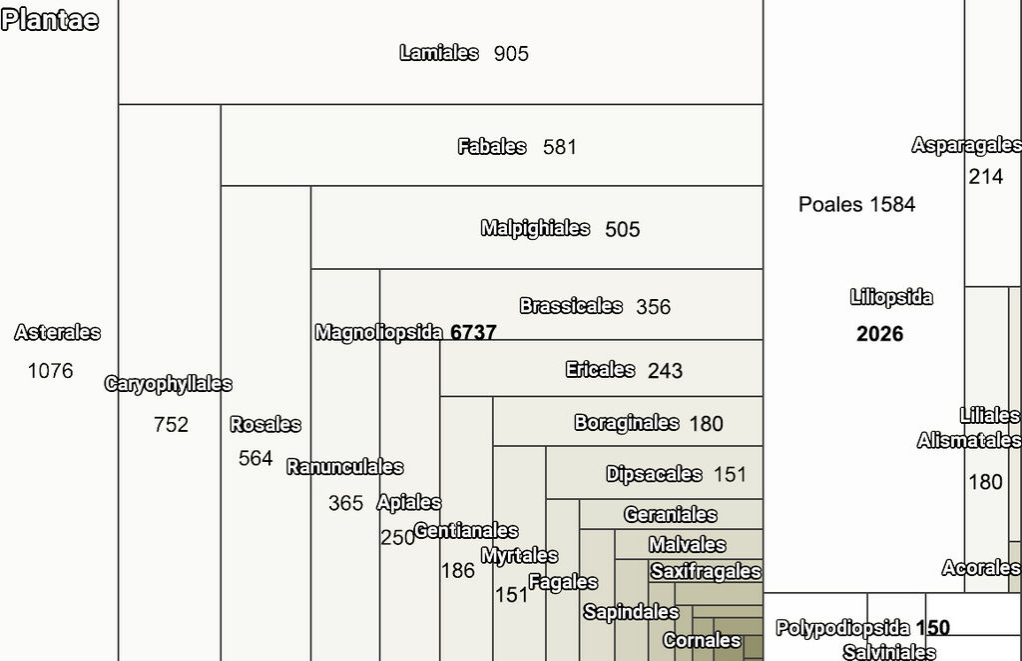
The leading classes and orders in vascular plant collections of the TUL Herbarium.

The digitised TUL collections of vascular plants contain 9,000 specimens which belong to 1,131 species and nothospecies (hybrids), 514 genera, 98 families, 40 orders and five classes. The correlation of represented orders is shown in Fig. 8 retrieved from GBIF webpage of our dataset. There are no type materials in the TUL Herbarium.

The most sampled families, genera and species of the TUL Herbarium are given in Table 2, Table 3 and Table 4.

TUL Herbarium documents hundreds of localities of rare and protected species of the region. Its collections were intensively used during the preparation of the "Red Data Book of Tula Oblast". In the current first edition of the Book (Shcherbakov 2010), there are 165 species of vascular plants. The updated second edition which is in preparation now includes 158 species, of which 129 are represented in the TUL Herbarium (Table 5).

At present, 29 of 158 legally-protected species of Tula Oblast are not represented in the TUL Herbarium. Most of them are kept in the Moscow University Herbarium (Seregin 2020b), as well as in some lesser herbaria of Tula. Some of these species are very rare or their populations are too small to afford collecting of at least a single individual. Nevertheless, one of the important tasks of the Herbarium is to replenish the collections with specimens of the missing species in a professional manner.

### Taxa included

**Table:** 

Rank	Scientific Name	
phylum	Tracheophyta	

## Traits coverage

### Data coverage of traits

PLEASE FILL IN TRAIT INFORMATION HERE

## Temporal coverage

### Notes

From 20 June 1919 to 26 October 2019.

A hundred years is the time interval between the oldest and the freshest specimens of the TUL Herbarium. The first one is a specimen of *Gentianella amarella* (L.) Börner collected by K.S. Dubensky on 20 June 1919 and the latter is a specimen of *Solidago canadensis* L. collected by I.S. Sheremetyeva on 26 October 2019 in line with the digitisation project.

**Figure 9.**
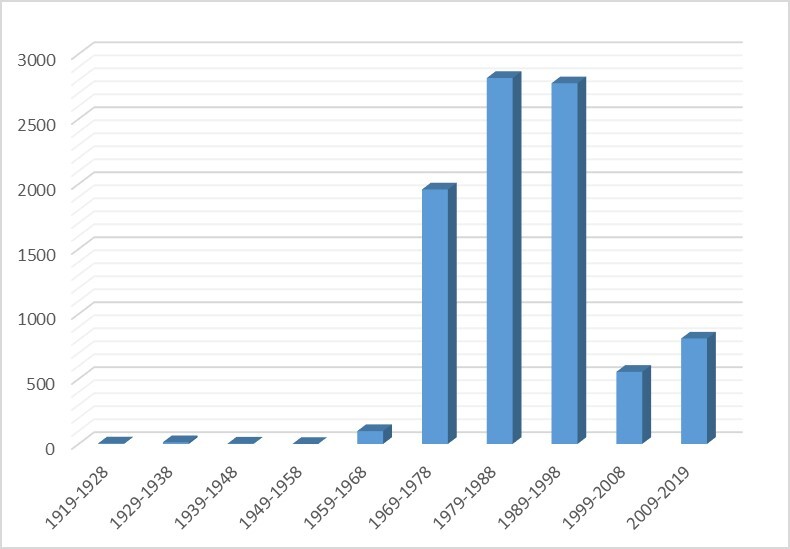
Temporal distribution of specimens in the TUL Herbarium

The temporal distribution of collected specimens over decades is given in Fig. 9.

A brief description of peaks and other significant dates is given below. Total figures are pointed as estimates because some specimens do not have an exact date, but very likely belong to this period.

**1919**–**1928**: Four specimens collected by K.S. Dubensky in 1919.

**1929**–**1938**: 13 specimens collected by V.A. Arsenyev in 1931.

**1939**–**1948**: Three specimens collected by A.I. Alyushin in 1939 (his first collections).

**1949**–**1958**: no collections.

**1959**–**1968**: 197 specimens, i.e. the first collections made in Tula State Lev Tolstoy Pedagogical Institute; also, five specimens collected by A.I. Alyushin.

**1969**–**1978**: 1,728 specimens collected by A.I. Alyushin and three by I.S. Sheremetyeva. Other specimens (ca. 300) are ordinary collections of students made during their summer field practices.

**1979**–**1988**: 2,816 specimens, incl. 710 collected by I.S. Sheremetyeva, 365 by A.I. Alyushin, 178 by E.G. Balashova and T.Yu. Svetasheva, 167 by L.V. Khoroon, 99 by E. Zenina, 80 by M. Andreeva, 71 by A.V. Shcherbakov and I.S. Sheremetyeva, 50 by E. Azarova and 43 by I. Blokhin. An interesting collection with 15 specimens was made by the famous Moscow Botanist V.E. Skvortsov together with I.S. Sheremetyeva in the Efremovsky District in 1988.

**1989**–**1998**: 2,775 specimens, incl. 777 collected by I.S. Sheremetyeva, 733 by L.V. Khoroon, 452 by E.G. Balashova and T.Yu. Svetasheva, 215 by A.V. Shcherbakov, I.S. Sheremetyeva and P.B. Sheremetyev, 130 by I. Dorokhina and 112 by E. Zenina. A small interesting collection with 32 specimens was made by I.S. Sheremetyeva together with the Moscow Botanists V.S. Novikov, N.B. Oktyabreva and K.V. Kiseleva in botanically well-known area of "Lupishki bogs" in 1990–1992. Other specimens are ordinary collections of students made during their summer field practices.

**1999**–**2008**: 546 specimens, incl. 271 collected by I.S. Sheremetyeva, L.V. Khoroon, T.Yu. Svetasheva and P.B. Sheremetyev made during research of protected areas in Tula Oblast. The results were published in the special edition of the "Red Data Book" devoted to the protected nature areas of Tula Oblast (Tararina et al. 2007). A total of 15 specimens were collected by E.M. Volkova, mainly on bogs.

**2009**–**2019**: 811 specimens, incl. 136 collected by I.S. Sheremetyeva (Sheremetyeva and Svetasheva 2019, Svetasheva and Sheremetyeva 2019, Svetasheva et al. 2019), 74 by T.Yu. Svetasheva assisted by A.F. Lakomov and M. Makarova, 46 by E.M. Volkova and 43 by M. Makarova. Other specimens are ordinary collections of students made during their summer field practices.

## Usage licence

### Usage licence

Other

### IP rights notes

This work is licensed under a Creative Commons Attribution (CC-BY) 4.0 License.

## Data resources

### Data package title

TUL Herbarium: Tula Oblast collections of vascular plants (https://doi.org/10.15468/ca08cm)

### Resource link

https://www.gbif.org/dataset/5788c83a-0e0a-42c1-8fcd-3b27ce7a672a

### Alternative identifiers

https://doi.org/10.15468/ca08cm; https://depo.msu.ru/ipt/resource?r=tula

### Number of data sets

1

### Data set 1.

#### Data set name

TUL Herbarium: Tula Oblast collections of vascular plants

#### Data format

Darwin Core

#### Number of columns

1

#### Description

TUL Herbarium presents in GBIF collections of vascular plants from Tula Oblast (9,000 specimens) imaged in December 2019 – January 2020 by the commercial partner (https://elar.ru/). The imaging was supported by the research project 19-44-710002 funded by RFBR and Tula Oblast Government. Databasing and georeferencing of the specimens from the TUL Herbarium were performed by staff members of the Tula State Lev Tolstoy Pedagogical University and Tula Local History Museum. The labels of all digitised specimens were fully databased and ca. 80% of specimens were georeferenced by May 2020. All data were published in the Moscow Digital Herbarium in 2019–2020 (https://plant.depo.msu.ru/) and are fully available in GBIF (Svetasheva and Seregin 2020).

**Data set 1.** 

Column label	Column description
TUL Herbarium: Tula Oblast collections of vascular plants	In 2019–2020, the comercial partner imaged the TUL Herbarium (Tula Oblast collections). In total, 9,000 specimens were digitised (300 dpi images and key metadata). These data were published in the Moscow Digital Herbarium in 2020 and are fully available in GBIF. Based on these data, a detailed overview of the physical collections is given in this data paper, as well as spatial, temporal and taxonomic description of the dataset. As of November 2020, 7,433 specimens from the TUL Herbarium have been georeferenced (82.6%).

## Additional information

**Collectors.** More than 700 collectors are listed in the database of the TUL Herbarium. The vast majority of them are students, but at least 20 are researchers. The top collectors with contribution of more than 70 specimens are given in Table 6.

**Dataset availability in Internet**

Svetasheva T, Seregin A (2019): TUL Herbarium: Tula Oblast collections of vascular plants. v 1.31. Tula State Lev Tolstoy Pedagogical University. Dataset/Occurrence. https://depo.msu.ru/ipt/resource?r=tula&v=1.31

Svetasheva T, Seregin A (2020). TUL Herbarium: Tula Oblast collections of vascular plants. Version 1.34. Tula State Lev Tolstoy Pedagogical University. Occurrence dataset https://doi.org/10.15468/ca08cm accessed via GBIF.org on 2020-09-28.

## Figures and Tables

**Figure 10.**
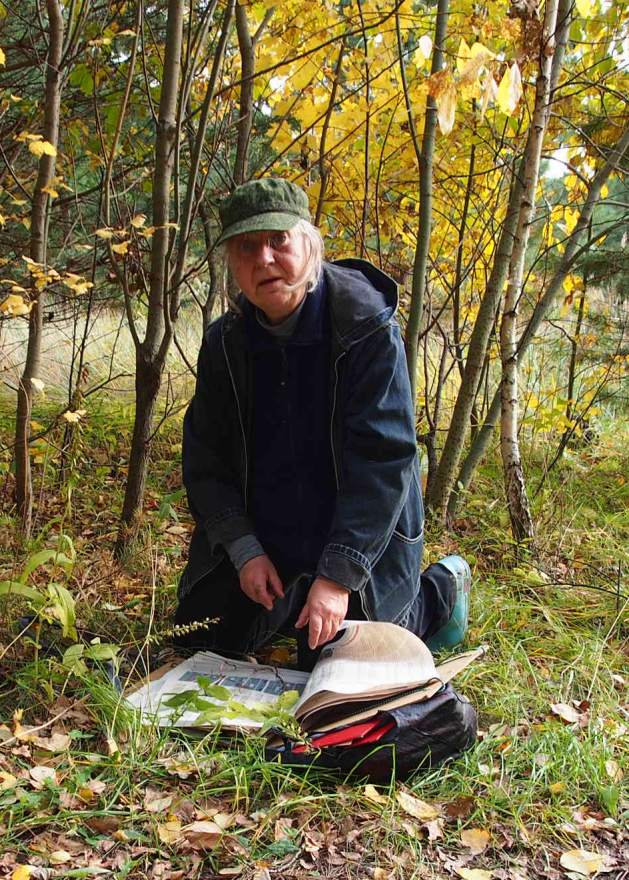
I.S. Sheremetyeva collecting plants on field excursion, 2019.

**Figure 11.**
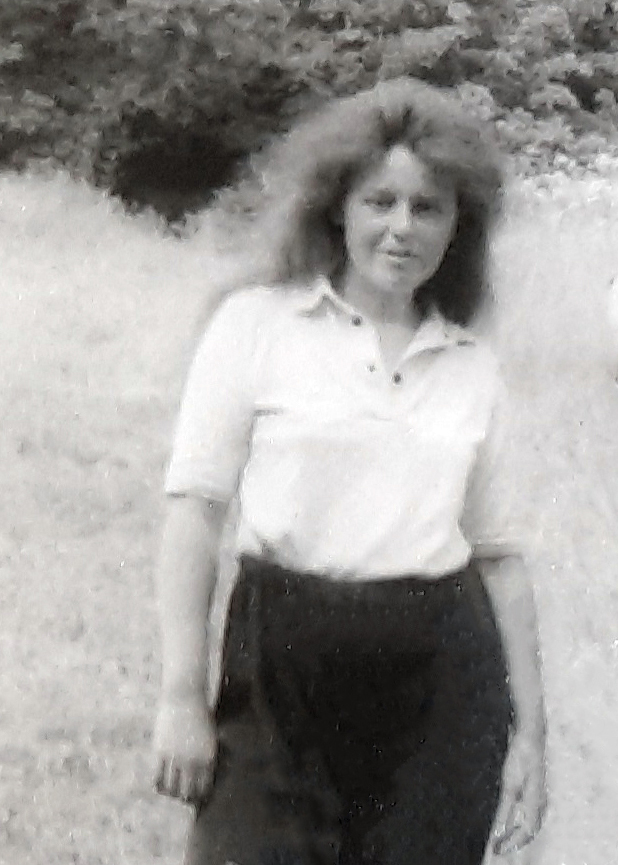
L.V. Khoroon at the field practice, 1991.

**Figure 12.**
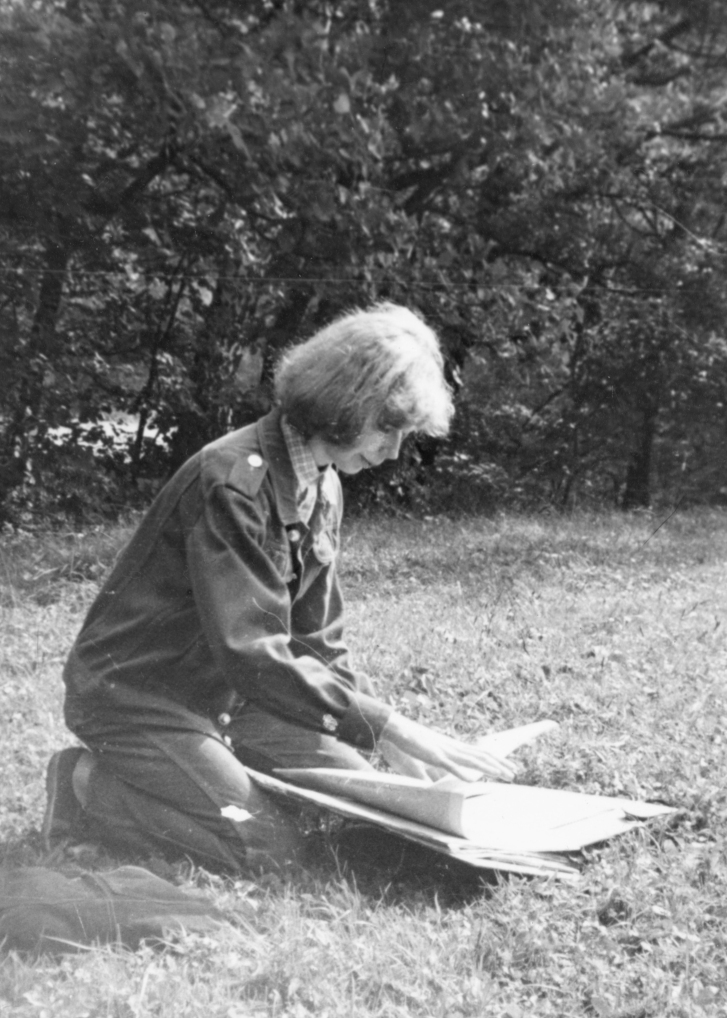
T.Yu. Svetasheva collecting plants on field excursion, 1989.

**Figure 13.**
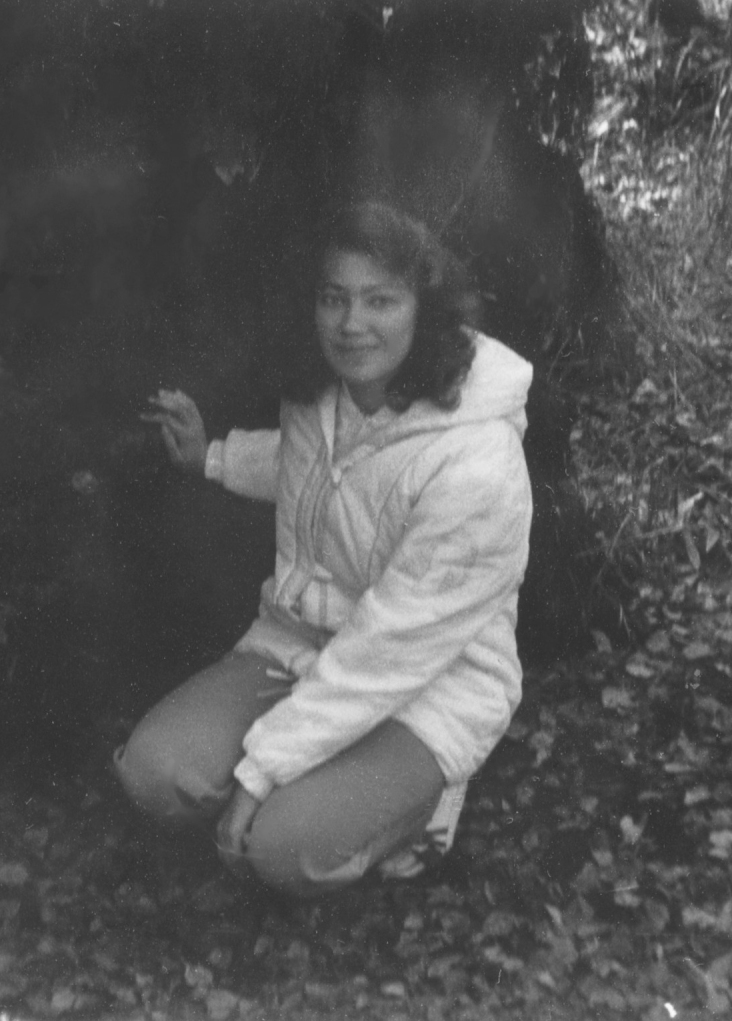
E.G. Balashova during field research, 1989.

**Figure 14.**
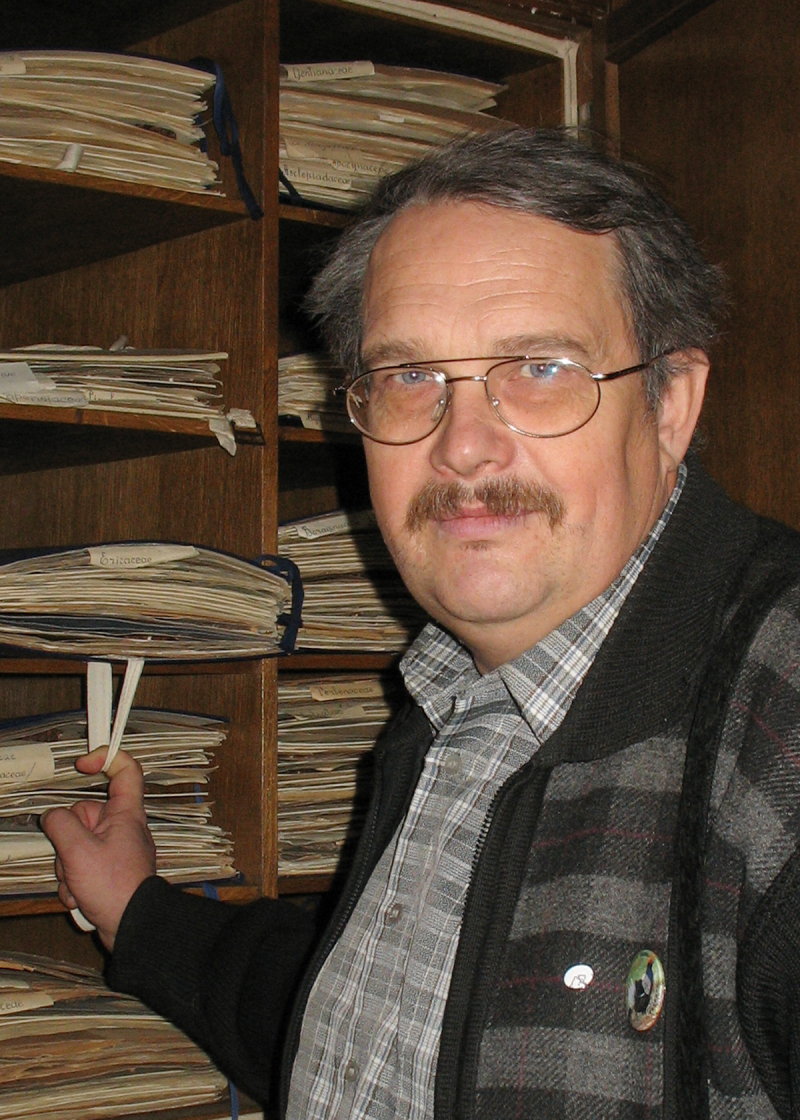
A.V. Shcherbakov at the Moscow University Herbarium, 2008.

**Figure 15.**
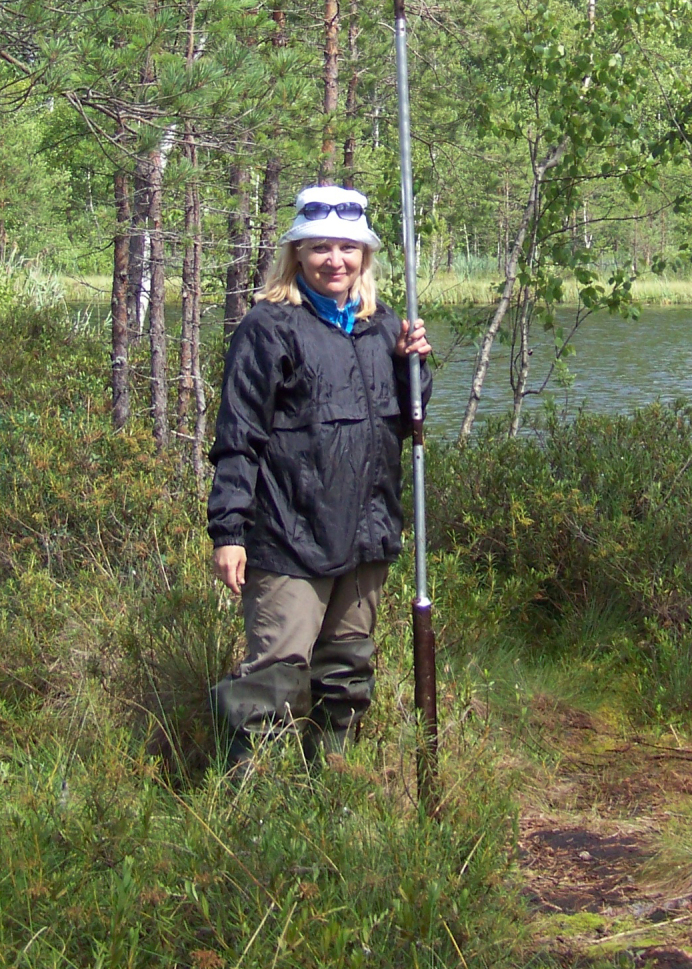
E.M. Volkova, during field research of the bog, 2007.

**Table 1.** Collections of the TUL Herbarium from administrative districts of Tula Oblast.

**Rank**	**First-level administrative unit (districts, cities)**	**Number of digitised specimens**	**Estimated total** **number of** **specimens**
1	Shchekinsky	1,682	1,734
2	Tula	1,334	1,386
3	Leninsky	715	740
4	Suvorovsky	711	739
5	Yasnogorsky	629	638
6	Efremovsky	500	517
7	Odoevsky	470	474
8	Kimovsky	389	415
9	Aleksinsky	321	324
10	Plavsky	271	285
11	Kurkinsky	267	277
12	Zaoksky	236	240
13	Dubensky	211	217
14	Uzlovskoy	208	214
15	Chernsky	177	179
16	Belevsky	120	127
17	Bogoroditsky	120	120
18	Venevsky	113	116
19	Teplo-Ogarevsky	92	93
20	Volovsky	80	84
21	Kireevsky	57	62
22	Novomoskovsky	55	59
23	Kamensky	46	47
24	Arsenyevsky	26	29

**Table 2.** The top families in vascular plant collections of the TUL Herbarium.

**Rank**	**Family**	**Number of specimens**	**Rank**	**Family**	**Number of** **specimens**
1	Asteraceae	931	11	Apiaceae	250
2	Poaceae	891	12	Plantaginaceae	247
3	Fabaceae	536	13	Salicaceae	226
4	Lamiaceae	531	14	Boraginaceae	173
5	Cyperaceae	514	15	Amaranthaceae	161
6	Rosaceae	504	16	Juncaceae	144
7	Brassicaceae	345	17	Campanulaceae	137
8	Caryophyllaceae	315	18	Ericaceae	133
9	Ranunculaceae	397	19	Rubiaceae	130
10	Polygonaceae	251	20	Caprifoliaceae	128

**Table 3.** The top genera in vascular plant collections of the TUL Herbarium.

**Rank**	**Genus**	**Number of** **specimens**	**Rank**	**Genus**	**Number of** **specimens**
1	* Carex *	389	11	* Stipa *	97
2	* Salix *	185	12	* Artemisia *	89
3	* Trifolium *	168	13	* Vicia *	85
4	* Veronica *	147	14	* Bromus *	84
5	* Galium *	126	15	* Rumex *	84
6	* Campanula *	122	16	* Lathyrus *	83
7	* Viola *	112	17	* Silene *	80
8	* Ranunculus *	109	18	* Epilobium *	79
9	* Juncus *	108	19	* Potamogeton *	75
10	* Potentilla *	103	20	* Euphorbia *	74

**Table 4.** The top species in vascular plant collections of the TUL Herbarium.

**Rank**	**Species**	**Number of** **specimens**	**Rank**	**Species**	**Number of** **specimens**
1	*Knautia arvensis* Coult.	44	11	*Carex nigra* Reich.	31
2	*Stipa pennata* L.	43	12	*Mentha arvensis* L.	31
3	*Euphorbia esula* L.	40	13	*Setaria viridis* (L.) P.Beauv.	29
4	*Salix cinerea* L.	39	14	*Thymus pulegioides* L.	29
5	*Prunella vulgaris* L.	37	15	*Persicaria lapathifolia* (L.) Gray	28
6	*Chenopodium album* L.	34	16	*Stipa capillata* L.	28
7	*Polygala comosa* Schkuhr	34	17	*Lotus corniculatus* L.	28
8	*Veronica teucrium* L.	33	18	*Achillea millefolium* L.	27
9	*Polygonum aviculare* L.	33	19	*Centaurea jacea* L.	27
10	*Juncus compressus* Jacq.	32	20	*Bistorta officinalis* Raf.	27

**Table 5.** The species included in the second edition of the "Red Data Book of Tula Oblast" (in press)

№	**Species**	**Number of specimens**
1	*Adenophora liliifolia* (L.) A.DC.	8
2	*Adonis vernalis* L.	25
3	*Allium flavescens* Besser	11
4	*Allium podolicum* Blocki ex Racib. & Szafer	6
5	*Alnus incana* (L.) Moench	14
6	*Andromeda polifolia* L.	1
7	*Anemone nemorosa* L.	4
8	*Anthericum ramosum* L.	14
9	Arabis planisiliqua subsp. nemorensis (Wolf ex Hoffm.) Soják [= *Arabis gerardii* (Bess.) Bess, ex Koch]	1
10	*Artemisia armeniaca* Lam.	4
11	*Artemisia latifolia* Ledeb.	15
12	*Artemisia sericea* Weber	14
13	*Asperula cynanchica* L	1
14	*Aster amellus* L	13
15	*Astragalus onobrychis* L.	2
16	*Betula humilis* Schrank	3
17	*Botrychium lunaria* (L.) Sw.	1
18	*Bupleurum falcatum* L.	7
19	*Calluna vulgaris* (L.) Hull	9
20	Campanula stevenii subsp. altaica (Ledeb.) Fed. [= *C. altaica* Ledeb.]	5
21	*Cardamine bulbifera* Crantz [= *Dentaria bulbifera* L.]	4
22	*Cardamine quinquefolia* (M.Bieb.) Schmalh. [= *Dentaria quinquefolia* L.]	10
23	*Carex appropinquata* Schumacher	6
24	*Carex atherodes* Spreng.	9
25	*Carex brunnescens* Poir.	5
26	*Carex dioica* L.	4
27	*Carex hartmanii* Cajander	2
28	*Carex lasiocarpa* Ehrh.	11
29	*Carex limosa* L.	5
30	*Carex michelii* Host	1
31	*Carex oederi* Retz. [= *C. serotina* Merat]	6
32	*Carex panicea* L.	10
33	*Carex vaginata* Tausch	3
34	*Chamaedaphne calyculata* Moench	6
35	*Circaea alpina* L.	3
36	*Cirsium canum* (L.) All.	7
37	*Cirsium pannonicum* Link	1
38	*Cladium mariscus* (L.) Pohl	8
39	*Clematis recta* L.	12
40	*Cotoneaster alaunicus* Golitsin	8
41	*Cypripedium calceolus* L.	5
42	*Daphne mezereum* L.	6
43	*Delphinium cuneatum* Steven ex DC.	7
44	*Dianthus borbasii* Vandas	3
45	Dianthus capitatus subsp. andrzejowskianus Zapal.	8
46	*Dianthus superbus* L.	9
47	*Diphasiastrum zeilleri* (Rouy) J. Holub [= *Lycopodium complanatum* L.]	4
48	*Dracocephalum ruyschiana* L.	6
49	*Drosera anglica* Huds.	11
50	*Drosera rotundifolia* L.	11
51	*Dryopteris expansa* (C.Presl) Fraser-Jenk. & Jermy	5
52	*Echinops ritro* L.	15
53	*Elymus lolioides* (P.Candargy) Melderis	5
54	*Epipactis palustris* Crantz	10
55	*Eriophorum vaginatum* L.	9
56	*Euphorbia palustris* L.	1
57	*Fritillaria meleagris* L.	10
58	*Galatella linosyris* Rchb.f.	7
59	*Gentiana pneumonanthe* L.	6
60	*Gladiolus imbricatus* L.	1
61	*Goodyera repens* R.Br.	3
62	*Gymnocarpium robertianum* Newm.	9
63	*Gypsophila altissima* L.	16
64	*Helianthemum nummularium* Mill.	11
65	*Helichrysum arenarium* Moench	26
66	*Helictochloa hookeri* (Scribn.) Romero Zarco [= *Helictotrichon schellianum* (Hack.) Kitagawa]	4
67	*Helictotrichon desertorum* (Less.) Pilg.	4
68	*Hepatica nobilis* Schreb.	6
69	*Huperzia selago* (L.) Bernh.	5
70	*Iris aphylla* L.	6
71	*Iris sibirica* L.	3
72	*Juniperus communis* L.	12
73	*Koeleria glauca* DC.	11
74	*Koeleria pyramidata* P.Beauv. [= *K. grandis* Bess ex Gorski]	1
75	*Laserpitium latifolium* L.	7
76	*Lathyrus palustris* L.	3
77	*Lilium martagon* L.	6
78	*Linum flavum* L.	8
79	*Linum perenne* L.	10
80	*Lunaria rediviva* L.	26
81	*Lycopodium clavatum* L.	23
82	*Melica transsilvanica* Schur	12
83	*Moneses uniflora* A.Gray	1
84	*Nymphaea candida* J.Presl & C.Presl	11
85	*Onosma simplicissimum* L.	6
86	*Orchis militaris* L.	1
87	*Ostericum palustre* Besser [= *Angelica palustris* (Bess.) Hoffm.]	3
88	*Oxytropis pilosa* DC.	5
89	*Pedicularis palustris* L.	2
90	*Phegopteris connectilis* (Michx.) Watt	4
91	*Poa remota* Forselles	7
92	*Polygala amarella* Crantz	3
93	*Polygala sibirica* L.	5
94	*Polystichum braunii* (Spenn.) Fée	5
95	*Populus nigra* L.	5
96	*Prunella grandiflora* (L.) Turra	16
97	*Prunus tenella* Batsch [= *Amygdalus nana* L.]	8
98	*Psephellus marschallianus* (Spreng.) K.Koch [= *Centaurea marschalliana* Spreng. incl. *C. sumensis* Kalenicz.]	8
99	*Pulsatilla patens* Mill	3
100	*Rhaponticoides ruthenica* (Lam.) M. V. Agab. & Greuter [= *Centaurea ruthenica* Lam.]	4
101	*Rhododendron tomentosum* Harmaja [= *Ledum palustre* L.]	9
102	*Rhynchospora alba* Vahl	2
103	*Rubus nessensis* W.Hall	2
104	*Sagina nodosa* (L.) Fenzl	2
105	*Salix lapponum* L.	5
106	*Salix myrtilloides* L.	6
107	*Salix rosmarinifolia* L.	11
108	*Salvia glutinosa* L.	1
109	*Salvinia natans* (L.) All.	8
110	*Scheuchzeria palustris* L.	2
111	*Scilla siberica* Andrews	5
112	*Scolochloa festucacea* Link	5
113	*Scorzonera hispanica* L.	9
114	*Scutellaria altissima* L.	6
115	*Scutellaria supina* L.	6
116	*Spinulum annotinum* (L.) A.Haines [= *Lycopodium annotinum* L.]	20
117	*Spiraea crenata* L.	21
118	*Stipa capillata* L.	28
119	*Stipa pennata* L.	43
120	*Stipa pulcherrima* K.Koch	11
121	*Stipa tirsa* Steven	15
122	*Tephroseris integrifolia* (L.) Holub [= *Senecio integrifolius* (L.) Clairv.]	1
123	*Trifolium lupinaster* L.	1
124	*Trollius europaeus* L.	7
125	*Vaccinium oxycoccos*	1
126	*Vaccinium myrtillus* L.	18
127	*Vaccinium uliginosum* L.	6
128	*Vicia pisiformis* L.	6
129	*Viola tanaitica* Grosset	1

**Table 6.** The top collectors of the TUL Herbarium.

**Rank**	**Collector (name, occupation)**	**Number of specimens** **(total / personally)**
1	I.S. Sheremetyeva – lecturer, researcher (Fig. 10)	2,914 / 1,591
2	A.I. Alyushin – teacher, researcher (Fig. 5)	2,104 / 2,100
3	L.V. Khoroon – student, researcher (Fig. 11)	940 / 668
4	T.Yu. Svetasheva – student, lecturer, researcher (Fig. 12)	745 / 76
5	E.G. Balashova – student (Fig. 13)	624 / 4
6	A.V. Shcherbakov – researcher from Moscow University (Fig. 14)	322 / 129
7	E.V. Zenina – student	244 / 169
8	I.L. Dorokhina – student	216 / 148
9	M. Andreeva – student	99 / 80
10	E.M. Volkova – student, lecturer, researcher (Fig. 15)	72 / 65
